# Source Apportionment and Health Risks of Toxic Metals in Groundwater from a Dual-Aquifer Coal Mine System

**DOI:** 10.3390/toxics14090740

**Published:** 2026-08-22

**Authors:** Gan Jiang, Kai Chen, Qimeng Liu

**Affiliations:** 1 School of Earth and Environment, Anhui University of Science and Technology, Huainan 232001, China; 2025200028@aust.edu.cn; 2 State Key Laboratory for Safe Mining of Deep Coal Resources and Environment Protection, Huainan 232001, China; 3 Key Laboratory of Mine Water Resource Utilization of Anhui Higher Education Institutes, Suzhou University, Suzhou 234000, China

**Keywords:** groundwater, hydrochemistry, heavy metal pollution, human health risk, source apportionment

## Abstract

Heavy metal contamination in mining-affected groundwater may pose potential risks to drinking water safety and human health. In this study, 25 groundwater samples, including 18 from Aquifer IV of the Cenozoic unconsolidated porous aquifer system and 7 from the Taiyuan Formation limestone karst aquifer, were collected from the Zhuxianzhuang Coal Mine, Huaibei Coalfield, China. The Heavy Metal Pollution Index (HPI), Metal Index (MI), Positive Matrix Factorization (PMF), and oral-ingestion health risk model were applied to assess metal contamination, source contributions, and human health risks. HPI values ranged from 8.0 to 37.5, with a mean of 21.5, indicating a low overall pollution level. In contrast, MI values suggested cumulative metal contamination, with higher mean values in Taiyuan Formation limestone karst groundwater (5.60) than in Aquifer IV groundwater (2.38). PMF identified two major factors, interpreted as a natural water–rock interaction source and a combined anthropogenic and mining-related source. Health risks were consistently higher in Taiyuan Formation limestone karst groundwater. Non-carcinogenic risks were higher for children and mainly controlled by As, whereas carcinogenic risks were higher for adults and mainly associated with Cr. These findings highlight the need for aquifer-specific monitoring and source control to reduce toxic metal exposure in coal mining areas.

## 1. Introduction

Groundwater is an important component of water resources, and its sustainable development and utilization have long been a major concern in both society and research [1,2,3,4]. In coal mining areas, groundwater scarcity is particularly prominent. In addition, long-term coal mining activities have induced a series of eco-environmental effects [5,6,7,8]. Heavy metals released from human activities and mining operations have become important contaminants in groundwater in mining areas [9,10,11,12,13]. Previous studies have shown that long-term consumption of groundwater with excessive heavy metal concentrations may lead to a range of health problems, including organ failure, gastrointestinal cancers, and even death [14,15,16]. Accordingly, effective groundwater-resource management in mining areas requires clear characterization of heavy-metal distributions, their sources, and the associated human health risks.

Previous research in coal-mining areas has extensively addressed both heavy-metal source apportionment and groundwater-quality assessment. For example, Hao et al. reported that As and Sb in groundwater were sensitive to the influence of human mining activities and successfully extracted three principal components of heavy metal pollution using principal component analysis (PCA) [1]. Liu et al. applied multivariate statistical methods, including PCA and correlation analysis, to distinguish natural processes from anthropogenic pollution factors in groundwater from a typical coal mining area in North China. Their results showed that the groundwater chemistry in the study area was jointly controlled by water–rock interaction, mainly carbonate dissolution, mining activities, agricultural inputs, domestic pollution, and precipitation–evaporation processes [17]. Ju et al. used the PMF model to apportion the sources of heavy metals in groundwater from the Sunan mining area and found that 30.3% of the heavy metals were derived from mineral dissolution, 28.2% from salinization, 26.3% from mine wastewater leaching, 8.0% from Fe–Mn minerals, and 7.2% from coal gangue [18]. Zhu et al. reported that the mean EWQI value of groundwater in the Dashu mining area of southwestern China was 92, with 30% of the samples classified as poor quality; however, the human health risks remained within acceptable levels [19]. Fu et al. identified the main sources of groundwater contamination in the Zezhou Basin using PCA, including weathering of mineralized strata, leakage from landfill liners, mining and industrial activities, and agricultural nitrogen fertilizers [20]. These studies provide important references for the prevention and control of heavy metal contamination in regional groundwater.

Although many studies have investigated heavy metal contamination and source apportionment in groundwater in mining areas, several limitations remain. First, in terms of source identification, principal component analysis, which is commonly used in previous studies, can qualitatively identify potential ion sources but cannot quantify the contribution of each source to heavy metal concentrations. Second, most existing studies have treated groundwater in mining areas as a single system, with limited attention paid to contamination differences among different aquifers. As a result, systematic comparisons of heavy metal contamination characteristics in multi-aquifer groundwater systems remain insufficient.

The Lianghuai mining area, which comprises the Huaibei and Huainan coalfields, is an important coal production base in China, where heavy metal contamination in groundwater is a prominent environmental issue. As a representative mine in the Lianghuai mining area, the Zhuxianzhuang Coal Mine faces considerable pressure on water supply, and groundwater is the main water source for local residents [21]. Nevertheless, groundwater at this mine remains insufficiently characterized with respect to heavy-metal concentrations, source contributions, and associated human health risks. The lack of such information limits the basis for sustainable groundwater development and utilization in the area. Therefore, this study applied multiple methods, including mathematical statistical analysis, the PMF source apportionment model, and health risk assessment, to investigate Aquifer IV groundwater from the Cenozoic unconsolidated porous aquifer system and the Carboniferous Taiyuan Formation limestone aquifer in the Zhuxianzhuang Coal Mine. The objectives were to: (1) identify the concentration characteristics and differences in heavy metals in groundwater from the two groundwater groups; (2) quantify the contributions of different sources to heavy metals in groundwater; and (3) assess the human health risks associated with heavy metals in groundwater. The results are expected to provide a reference for groundwater resource utilization and heavy metal pollution prevention and control in the study area and the broader Lianghuai mining area.

## 2. Materials and Methods

### 2.1. Overview of the Study Area

#### 2.1.1. Geographic and Climatic Conditions

The Zhuxianzhuang Coal Mine is located 13 km southeast of Suzhou City (under the jurisdiction of Yongqiao District) and 64 km northwest of Huaibei City, China. Its geographical coordinates lie between 117°05′37.6″–117°09′23″ E and 33°33′31.4″–33°39′37.6″ N. The region experiences a mild, warm-temperate, semi-humid monsoon climate characterized by four distinct seasons, with a long-term average annual precipitation of 883.7 mm.

#### 2.1.2. Hydrogeological Conditions

**Figure 1:**
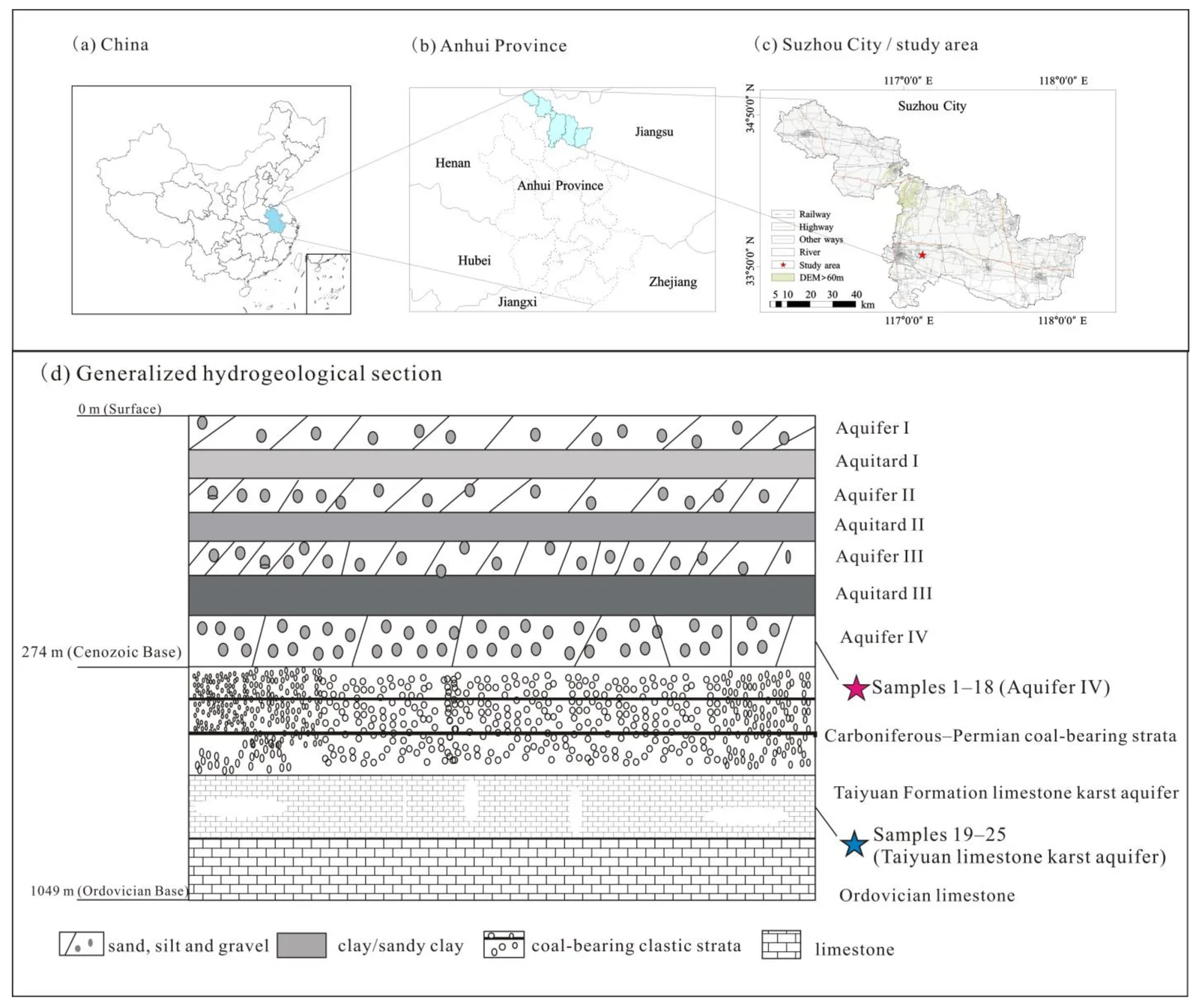
Regional location and generalized hydrogeological section of the study area. Note: (**a**) China; (**b**) Anhui Province; (**c**) Suzhou City and the study area; (**d**) generalized hydrogeological section. Panel (**d**) is not to scale.

The spatial distribution of the aquifers in the study area is depicted in Figure 1. According to borehole core logging, the hydrogeological profile is vertically divided into three primary aquifer systems: the Cenozoic unconsolidated porous aquifer system, the bedrock roof and coal-measure fractured aquifer system, and the bedrock karst aquifer system.

The “Cenozoic unconsolidated porous aquifer system” comprises Quaternary and Neogene argillaceous silt, fine sand, and sandy clay. Based on burial depth, it is subdivided into four aquifers (Aquifers I to IV). The Cenozoic unconsolidated sequence is 211.85–273.76 m thick (mean 246.60 m). The bottom depths of Aquifers I–IV are 23.82–56.30 m (mean 33.20 m), 65.35–94.85 m (mean 83.20 m), 100.57–167.00 m (mean 132.30 m), and 211.85–273.76 m (mean 246.60 m), respectively. The third aquitard has a bottom depth of 194.39–253.70 m and a thickness of 54.66–113.37 m (mean 89.90 m). It is composed mainly of sandy clay, clay, and calcareous clay and is characterized by low permeability and good confining capacity. All 18 groundwater samples analyzed from the Cenozoic unconsolidated porous aquifer system were collected from Aquifer IV; therefore, the following comparison specifically represents Aquifer IV groundwater rather than a mixture of Aquifers I–IV. The “bedrock roof and coal-measure fractured aquifer system” primarily consists of sandstone fractured aquifers within the coal-bearing strata. These sandstone fractured aquifers are generally separated by mudstone and siltstone and remain relatively independent under normal conditions, although water-conducting faults can locally enhance their hydraulic connectivity. The “bedrock karst aquifer system” is predominantly composed of the Carboniferous Taiyuan Formation limestone and the underlying Ordovician limestone. Karst development in the Taiyuan limestone aquifer is highly heterogeneous, constituting the primary threat for floor water inrushes. The Carboniferous Taiyuan Formation is approximately 140 m thick and contains 11–12 limestone beds with a cumulative limestone thickness of about 62 m. Its hydraulic properties are spatially and vertically heterogeneous. In shallow or Level I sections, specific capacity ranges from 0.04573 to 0.9039 L/(s·m), and hydraulic conductivity ranges from 0.1843 to 5.5836 m/d. In contrast, the deeper Level II–III sections are generally weakly water-bearing, with specific capacity of 0.0000152–0.012 L/(s·m) and hydraulic conductivity of 0.0000052–0.076 m/d. The mining license covers elevations from −250 to −1100 m, and the mine is divided into Level I (−250 to −435 m), Level II (−435 to −700 m), and Level III (−700 to −1000 m). Mine monitoring records indicate little change in Taiyuan Formation groundwater levels in northern areas with limited nearby drainage or mining, whereas the southern area affected by underground drainage experienced an average water-level decline of 157.83 m. Under mining and drainage disturbances, faults and structural fractures, mining-induced fractures, floor fractures, and water-conducting boreholes may provide local preferential hydraulic connections. These features represent plausible hydrogeological mechanisms by which the deep Taiyuan limestone groundwater may be influenced externally.

### 2.2. Sample Collection and Laboratory Analysis

Groundwater samples were collected following the Technical Specifications for Environmental Monitoring of Groundwater (HJ 164-2020) [22]. A total of 25 groundwater samples were collected, including 18 samples from Aquifer IV of the Cenozoic unconsolidated porous aquifer system and 7 samples from the Carboniferous Taiyuan Formation limestone karst aquifer. Aquifer IV samples were obtained using water pumps. Before sample collection, each well was purged for 20 min to allow the in situ water temperature, pH, and electrical conductivity to stabilize. Taiyuan Formation limestone karst groundwater samples were collected directly from the discharge of underground drainage boreholes in mining roadways during roadway development.

Each groundwater sample was filtered in the field with a 0.45 μm membrane and then stored in a 500 mL high-density polyethylene bottle that had previously been soaked in 10% nitric acid and rinsed with ultrapure water. For trace-element analysis, the samples were acidified with high-purity nitric acid to pH < 2, maintained at 4 °C in a cooler during transport, and analyzed within one week.

Inductively coupled plasma mass spectrometry(ICP-MS; ICPMS-2030LF, Shimadzu, Kyoto, Japan) was used to quantify 16 elements: V, Cr, Co, Cu, Zn, As, Rb, Mo, Sn, Sb, Pb, U, Mn, Fe, Sr, and Ba. Analytical quality assurance and quality control were evaluated using procedural blanks, duplicate samples, and matrix spike recovery tests. Duplicate analysis was performed at a frequency of one sample per ten samples, and all relative percent differences were below 5%. For the target elements, matrix spike recoveries were between 95% and 105%. Method detection limits were obtained using the three-standard-deviation method [23].

### 2.3. Comprehensive Water Quality Assessment Methods

The Heavy Metal Pollution Index (HPI) and Metal Index (MI) were applied to characterize heavy metal contamination and assess the suitability of groundwater for drinking purposes.

#### 2.3.1. Heavy Metal Pollution Index (HPI)

The Heavy Metal Pollution Index (HPI) combines weighted scores for individual metal elements to provide an integrated measure of heavy metal pollution in water [24,25,26,27]. The index was calculated as follows:

(1) $$\frac{\sum_{i=1}^{n}w_{iQ_{i}}}{\sum_{i=1}^{n}W_{i}}$$

where *W_i_* denotes the weight assigned to the *i*-th element and equals the reciprocal of its standard limit (*S_i_*). *Q_i_* denotes the sub-index of the *i*-th parameter and was calculated as:

(2) Qi=(CiSi)×100

An HPI value of 100 is commonly adopted as the threshold indicating heavy metal pollution [28].

#### 2.3.2. Metal Index (MI)

Because the weighting and averaging procedure used in HPI can reduce the apparent influence of some high-concentration elements, the Metal Index (*MI*) was additionally applied to evaluate groundwater suitability for drinking. Unlike HPI, MI represents the combined pollution effect of multiple heavy metals on water quality and was calculated as:

(3) $$MI=\sum_{i=1}^{n}\frac{C_{i}}{S_{i}}$$

where *C_i_* denotes the measured concentration of the *i*-th heavy metal, while *S_i_* denotes the corresponding standard limit.

Based on the *MI* value, water-quality contamination was grouped into six categories:

*MI* ≤ 0.3: Very pure

0.3 < *MI* ≤ 1.0: Pure

1.0 < *MI* ≤ 2.0: Slightly affected

2.0 < *MI* ≤ 4.0: Moderately affected

4.0 < *MI* ≤ 6.0: Strongly affected

*MI* > 6.0: Seriously affected

Groundwater with *MI* > 1.0 was regarded as exceeding the warning level for drinking-water quality.

#### 2.3.3. Specification of Evaluation Standards

In the calculation of the evaluation indices, the standard limits (S_i_) for individual elements were preferentially adopted from the Class III thresholds of the Chinese Standard for Groundwater Quality (GB/T 14848-2017) [29]. For trace elements not included in this standard, supplementary standards or guideline values were used. The standard limit for uranium (U) was taken from the World Health Organization (WHO) Guidelines for Drinking-water Quality [30] (30 μg/L). The limit for strontium (Sr) was based on the health advisory level of the United States Environmental Protection Agency (USEPA) [31] (4000 μg/L). The limit for vanadium (V) was adopted from the specific parameter limit in the Chinese Environmental Quality Standards for Surface Water (GB 3838-2002) [32] (50 μg/L). Rubidium (Rb) and tin (Sn) were excluded from the HPI and MI calculations because no corresponding drinking water standard limits are available in relevant Chinese or international guidelines.

### 2.4. Positive Matrix Factorization (PMF) Source Apportionment

Source apportionment was conducted with the Positive Matrix Factorization model developed by the United States Environmental Protection Agency (USEPA PMF 5.0) [33]. In PMF, the receptor concentration matrix (X) is resolved into a sample contribution matrix (G) and a source profile matrix (F). The matrix relationship is written as follows:

(4) $$X_{ij}=\sum_{k=1}^{p}g_{ik}f_{kj}+e_{ij}$$

where *X_ij_* denotes the measured concentration of element *j* in sample *i*; *g_ik_* denotes the contribution of source *k* to sample i; *f_kj_* represents the abundance of element j in source k; and *e_ij_* denotes the residual.

PMF uses the concentration matrix together with a corresponding uncertainty matrix as model inputs. For each element, uncertainty was estimated from the measured concentration (*C*) and its method detection limit (*MDL*):

(5) C≤MDL:                                                     Unc=56×MDL

(6) $${C>MDL:\;\;\;\;\;\;\;\;\;\;\;\;\;\;\;\;\;\;\;\;\;U}_{nc}=\sqrt{{\left(0.1\times C\right)}^{2}+{\left(0.5\times MDL\right)}^{2}}$$

In this calculation, the constant 0.1 corresponds to an assumed analytical error fraction of 10%.

During model optimization, variables were categorized on the basis of their signal-to-noise ratio (S/N). Pb, Sb, Fe, and Sr, which showed comparatively weaker stability, were designated as “Weak” variables. Given the limited sample size (*n* = 25), preliminary PMF trials using all 16 measured elements did not yield a sufficiently stable and reproducible factor solution. Therefore, the final source-apportionment model was restricted to the 10-species set that provided stable model performance and physically interpretable factor profiles; V, Mo, U, Rb, and Sn were not retained in the final PMF solution. The final PMF model included 10 species: As, Pb, Sb, Cr, Mn, Fe, Ba, Cu, Zn, and Sr. After comparing Q values, residual distributions, and factor interpretability across repeated trials, a two-factor solution was selected as optimal. The model was run 20 times to assess and improve the stability and reliability of the solution. An additional two-factor sensitivity analysis was performed by reintroducing Co while keeping the remaining model settings unchanged.

### 2.5. Human Health Risk Assessment

Potential health risks to local residents from heavy metals in groundwater were evaluated quantitatively using the health risk assessment framework recommended by the United States Environmental Protection Agency (USEPA). Because groundwater is the principal drinking water source in the study area, oral ingestion was considered the primary exposure route. Risks were evaluated separately for children and adults.

#### 2.5.1. Exposure Dose Estimation

For carcinogenic risk assessment, the average daily dose (*ADD*, mg/(kg·d)) associated with oral ingestion of heavy metals was estimated as follows:

(7) $$ADD=\frac{C_{\omega }\times IR\times EF\times ED}{BW\times AT}$$

where *C_ω_* denotes the measured concentration of the heavy metal in water (mg/L); *IR*, *EF*, *ED*, *BW*, and *AT* represent the daily water ingestion rate (L/d), exposure frequency (days/year), exposure duration (years), average body weight (kg), and averaging time (days), respectively. Exposure-parameter values were drawn mainly from USEPA guidance and the Exposure Factors Handbook of the Chinese Population and are provided in Table 1 [34,35].

#### 2.5.2. Characterization of Non-Carcinogenic and Carcinogenic Risks

(1) Non-carcinogenic Risk:

For an individual heavy metal, non-carcinogenic risk was characterized by the hazard quotient (*HQ*), whereas the combined non-carcinogenic risk from multiple metals was represented by the hazard index (*HI*) [36,37,38,39]. These indices were calculated as follows:

(8) $$HQ_{i}=\frac{C_{\omega }\times IR}{BW\times RfD_{i}}$$

(9) $$HI=\sum_{i=1}^{n}{HQ}_{i}$$

where *RfD_i_* denotes the oral reference dose (mg/(kg·d)) for element i. HQ or HI values below 1 were regarded as acceptable, whereas values above 1 indicate a potential non-carcinogenic health risk. For the non-carcinogenic risk assessment, the daily water ingestion rates were 0.7 L/d for children and 1.5 L/d for adults, and the corresponding body weights were 16.2 and 60.6 kg, respectively. The *RfD* values of all elements included in the non-carcinogenic risk assessment are provided in Table S1.

(2) Carcinogenic Risk:

On the basis of available toxicological parameters and the International Agency for Research on Cancer (IARC) classification, the carcinogenic risk assessment was limited to As and Cr, both recognized as carcinogenic. Single-element carcinogenic risk (CR) and total carcinogenic risk (TCR) were determined as follows:

(10) $${\mathrm{CR}}_{i}={ADD}_{i}\times {\mathrm{SF}}_{i}$$

(11) $$\mathrm{TCR}=\sum_{i=1}^{n}{CR}_{i}$$

where *SF_i_* denotes the oral slope factor ((kg·d)/mg) for carcinogenic element i. In the carcinogenic *ADD* calculation, the averaging time (*AT*) was taken as the life expectancy (70 years × 365 days = 25,550 days). For children and adults, the ingestion rates were 1.0 and 2.0 L/d, the corresponding body weights were 15 and 70 kg, and the exposure durations were 6 and 30 years, respectively; the exposure frequency was 350 days/year for both groups. Risk levels were interpreted according to USEPA guidance: CR or TCR values below 10^−6^ indicate negligible risk, values from 10^−6^ to 10^−4^ indicate acceptable risk, and values above 10^−4^ indicate unacceptable carcinogenic risk.

Table 1 summarizes the exposure parameters used in the non-carcinogenic and carcinogenic risk assessments, whereas the element-specific toxicological parameters are provided separately in Table S1. Oral reference doses (RfDs) were used exclusively for the calculation of non-carcinogenic risks (HQ and HI), while oral cancer slope factors (SFs) were used for carcinogenic risks (CR and TCR). The non-carcinogenic assessment included 14 trace elements, and the corresponding RfD values are listed in Table S1 together with their respective sources. Carcinogenic risks were evaluated for As and Cr using SF values of 1.5 and 0.5 (mg/(kg·day))^−1^, respectively [40]. Because chromium speciation was not determined, the measured total Cr concentrations were conservatively assessed using the toxicity parameters for Cr (VI).

## 3. Results and Discussion

### 3.1. Distribution Characteristics of Heavy Metals in Mine Groundwater

**Figure 2:**
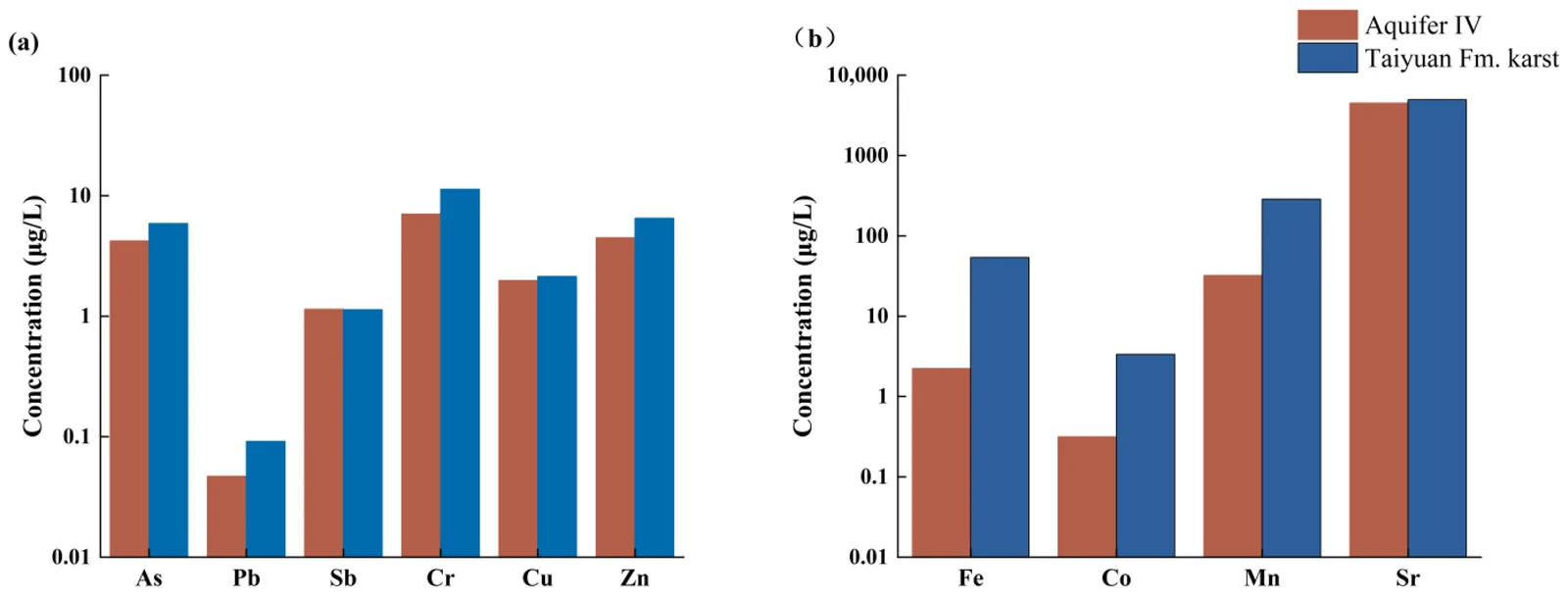
Mean concentrations of ten selected elements in Aquifer IV groundwater and Taiyuan Formation limestone karst groundwater: (**a**) As, Pb, Sb, Cr, Cu, and Zn; (**b**) Fe, Co, Mn, and Sr.

The concentration characteristics of the 16 measured elements are summarized in Table 2, while Figure 2 presents ten selected elements to provide a readable comparison between Aquifer IV groundwater and Taiyuan Formation limestone karst groundwater. The selection was designed to cover the two types of signals emphasized in the subsequent interpretation: As, Pb, Sb, Cr, Cu, and Zn are discussed primarily in relation to anthropogenic/mining-related inputs, whereas Fe, Co, Mn, and Sr were included to illustrate hydrogeochemical contrasts between the two groundwater groups. Sample-level concentrations for all 16 elements are provided in Table S2. The mean concentrations of most elements were lower than the Class III limits of the Chinese Standard for Groundwater Quality (GB/T 14848-2017) [29] and the WHO drinking water guidelines [30], indicating generally good groundwater quality in the study area.

As shown in Table 2, only Mn and Sr exceeded the corresponding standard limits in some samples. Based on the concentration ranges in the two groundwater groups, the six samples exceeding the Mn limit were all from the Taiyuan Formation limestone karst groundwater, whereas only one Aquifer IV sample did not exceed the Sr limit.

In terms of mean concentrations, the elements in Aquifer IV groundwater followed the order: Sr > Mn > Rb > Ba > Cr > Zn > As > Fe > V > Cu > Mo > Sb > U > Co > Pb > Sn. In Taiyuan Formation limestone karst groundwater, the order was: Sr > Mn > Fe > Ba > Rb > Cr > Zn > As > V > Co > Mo > Cu > U > Sb > Pb > Sn. Overall, the mean concentrations of most elements in Taiyuan Formation limestone karst groundwater were higher than those in Aquifer IV groundwater, suggesting that Taiyuan Formation limestone karst groundwater was more affected by heavy metal contamination than Aquifer IV groundwater. This difference was also reflected in the mean concentration ratios between the two groundwater groups. The ratios were particularly high for Fe, Co, and Mn, reaching 24.23, 10.63, and 8.89, respectively.

The coefficient of variation (CV) was used to characterize the dispersion of elemental concentrations. In environmental source-apportionment studies, relatively high CV values are often associated with stronger spatial heterogeneity and possible anthropogenic influence. However, for chemical constituents in deep groundwater, high CV values may also reflect differences in the type and intensity of water–rock interaction [4,41]. In this study, CV values of <0.2, 0.2–0.5, and >0.5 were interpreted as low, moderate, and high variability, respectively. Accordingly, a high CV was taken to indicate marked spatial heterogeneity that may reflect local point-source inputs or changes in the geochemical environment. As shown in Table 2, Fe (CV = 0.71), Sb (CV = 0.73), U (CV = 0.80), and Sn (CV = 0.91) showed high variability in Aquifer IV groundwater. In Taiyuan Formation limestone karst groundwater, Mn (CV = 0.72), Fe (CV = 1.06), and Sn (CV = 1.80) showed high variability. Fe had relatively high CV values in both groundwater groups, indicating that its sources may be complex and spatially variable.

### 3.2. Groundwater Pollution Assessment

**Figure 3:**
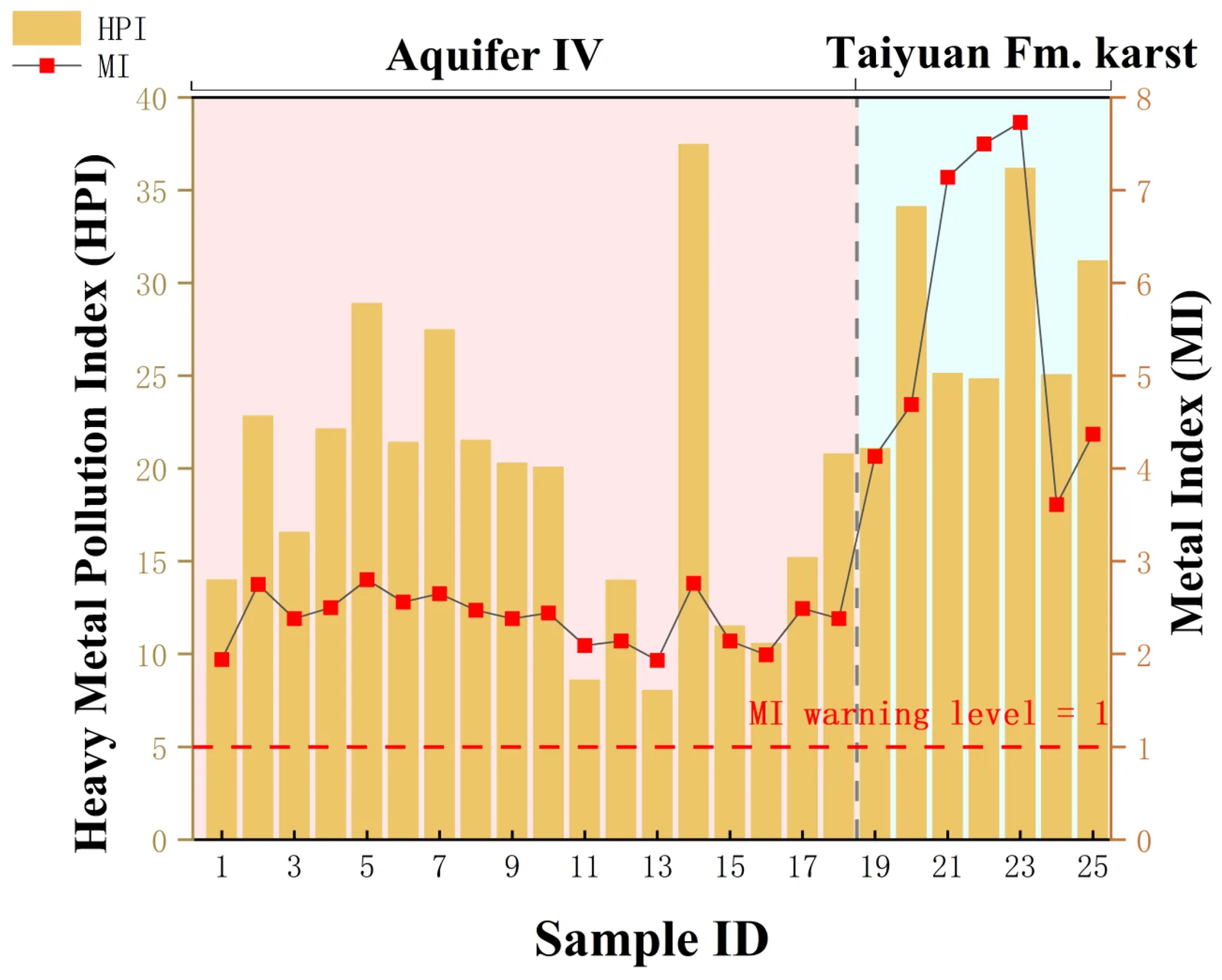
HPI and MI values of groundwater samples from Aquifer IV and the Taiyuan Formation limestone karst aquifer.

The heavy metal pollution levels in groundwater from the two groundwater groups were evaluated using the HPI and MI indices, and the results are shown in Figure 3. Across the 25 groundwater samples, HPI ranged from 8.03 to 37.45 and averaged 21.54. All HPI values remained far below the pollution threshold of 100, indicating that the overall heavy metal concentrations in groundwater remained within the safe range.

In comparison, the MI focuses on the cumulative pollution effects of multiple heavy metals on water quality [42,43,44,45]. As shown in Figure 3, every sample had an MI above 1, and the overall mean was 3.28, demonstrating a cumulative heavy metal pollution effect in groundwater across the study area. Further analysis showed that the Aquifer IV groundwater samples (Nos. 1–18) had a mean MI value of 2.38 and were mainly classified as slightly affected to moderately affected (1.0 < MI ≤ 4.0). In contrast, the Taiyuan Formation limestone karst groundwater showed a higher mean MI value of 5.60, corresponding to the strongly affected category. Taken together, these results show that the heavy metal pollution level in Aquifer IV groundwater was lower than that in the Taiyuan Formation limestone karst groundwater.

### 3.3. Source Apportionment via the PMF Model

**Figure 4:**
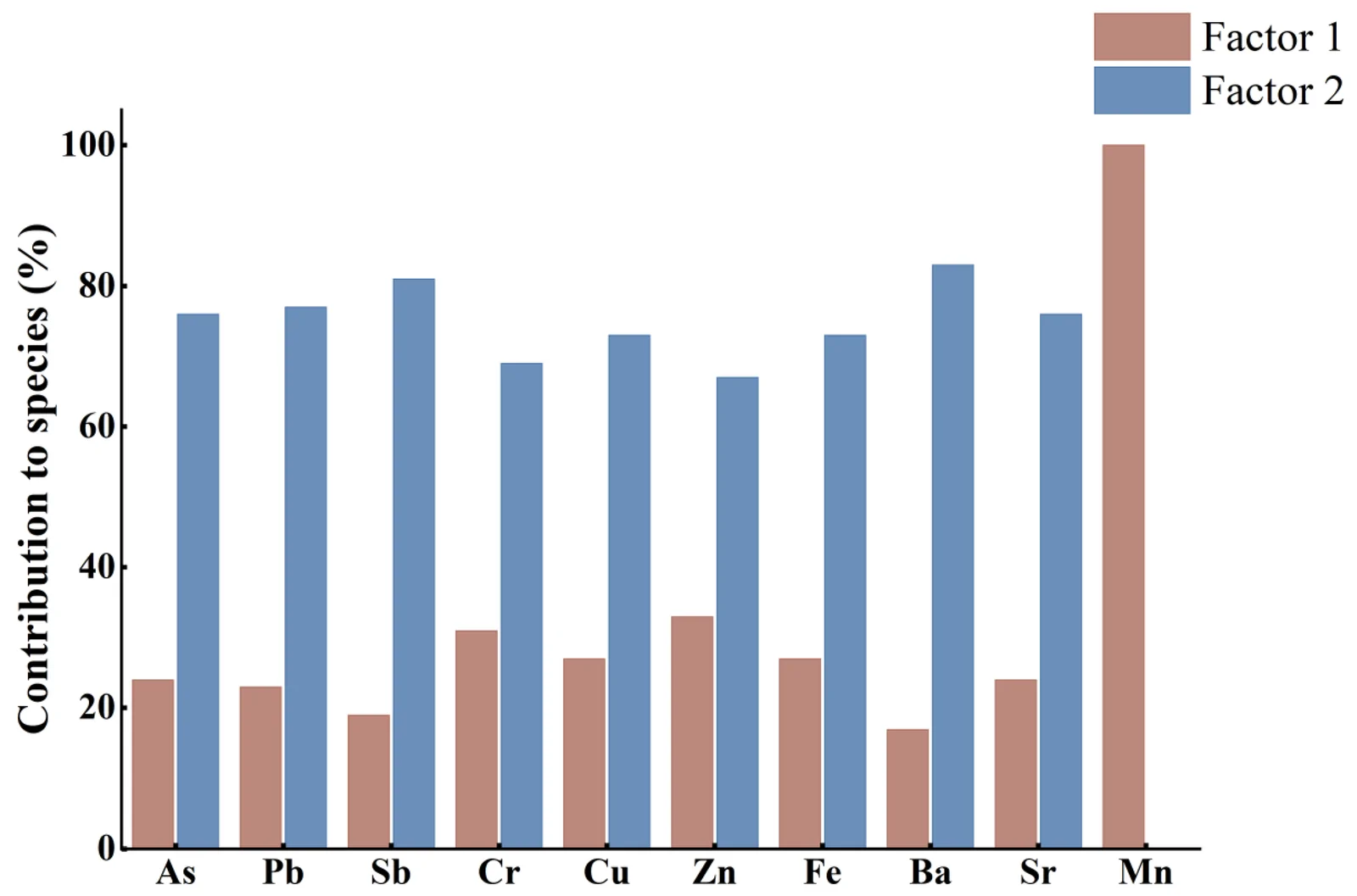
Species-specific relative contributions of Factor 1 and Factor 2 in the retained 10-species two-factor PMF solution.

To quantitatively distinguish the effects of natural geological background and anthropogenic mining activities on heavy metals in groundwater, the USEPA PMF 5.0 model was applied to 10 selected representative elements, including As, Pb, Sb, Cr, Mn, Fe, Ba, Cu, Zn, and Sr. All 20 base runs of the retained 10-species model converged and yielded nearly identical Q (Robust) and Q (True) values (approximately 651.9 and 680.1, respectively). The coefficients of determination (R^2^) for As, Cr, Mn, and Ba were greater than 0.70. In the Co sensitivity analysis, all 20 runs also converged; however, alternative factor solutions occurred across runs, Co showed a low model fit (R^2^ = 0.146), and the R^2^ for Mn decreased from 0.988 in the retained model to 0.622 after Co was included. Therefore, the more stable and interpretable 10-species solution was retained for source interpretation. The contribution profiles of the extracted factors are shown in Figure 4.

Factor 1 (Natural Water-Rock Interaction Source): As shown in Figure 4, Factor 1 was characterized by a high contribution of Mn, close to 100%, whereas the contributions of other elements were relatively low, generally below 35%. In groundwater environments, Mn is widely regarded as a sensitive geochemical indicator of aquifer redox evolution and variations in the primary geological environment [46]. Ba and Sr may have dual sources. On the one hand, as alkaline earth elements commonly associated with calcium-bearing minerals, they can enter groundwater through the weathering and leaching of carbonate and sulfate rocks [47]. On the other hand, when they show high contributions together with heavy metals such as As, Pb, and Cr, they may serve as tracers of industrial activities, including mining and chemical production, or surface water–groundwater interaction affected by anthropogenic inputs [48]. In Factor 1, the contribution of Mn was dominant, whereas the contributions of Ba and Sr were relatively low. Therefore, Factor 1 was interpreted as a natural geogenic source related to water–rock interaction and the hydrochemical background of the aquifer.

Factor 2 (Combined anthropogenic and mining-related source): In contrast to Factor 1, Factor 2 was mainly associated with typical exogenous heavy metals, including As, Pb, Sb, Cr, Cu, and Zn, with contributions of 76.2%, 77.6%, 81.3%, 69.7%, 73.6%, and 67.3%, respectively. In mine groundwater systems, elevated levels of Cu, Zn, Pb, and Cr may be related to the wear of underground machinery, transportation activities in mining areas, and agricultural inputs [49,50,51]. In addition, Cr, Sb, and Pb can be released through long-term leaching from coal gangue piles, infiltration associated with oxidative weathering of coal-bearing materials, and discharge of mining-related wastewater [52,53]. Ali et al. reported that irrigation-induced leaching can be an important source of As enrichment in groundwater [54]. Therefore, Factor 2 was interpreted as a combined anthropogenic and mining-related source associated with coal mining, industrial activities, and agricultural inputs.

Overall, the groundwater in the study area showed different source characteristics between the two groundwater groups. The sample-level G matrix showed that the mean normalized contribution of Factor 1 was substantially higher in the Taiyuan Formation limestone karst groundwater (2.738) than in Aquifer IV groundwater (0.321), whereas Factor 2 showed a higher mean normalized contribution in Aquifer IV groundwater (1.124) than in the Taiyuan Formation limestone karst groundwater (0.677) (Figure S1). Consistent with these results, Aquifer IV groundwater was mainly influenced by Factor 2, whereas the Taiyuan Formation limestone karst groundwater showed a stronger Factor 1 contribution together with continued influence from Factor 2. As discussed above (Table 2), the mean Mn concentration in Aquifer IV groundwater was 32.19 μg/L, which was below the recommended limit of 100 μg/L. Combined with the PMF results, Mn in Aquifer IV groundwater was mainly derived from Factor 1, suggesting relatively weak water–rock interaction in the Aquifer IV groundwater. Meanwhile, the CV values of As, Cr, and Pb, which are associated with anthropogenic industrial and agricultural activities, were 0.33, 0.13, and 0.49, respectively, indicating relatively uniform anthropogenic infiltration in the Aquifer IV groundwater.

In the deeper Taiyuan Formation limestone aquifer, the mean concentrations of Mn and Fe reached 286.06 and 53.96 μg/L, respectively, which were 8.89 and 24.23 times higher than those in the Aquifer IV groundwater. This suggests more intensive water–rock interaction in the Taiyuan Formation limestone karst aquifer. Compared with the Aquifer IV groundwater, the concentrations of As, Cr, and Pb, which are related to anthropogenic industrial and agricultural activities, also increased to varying degrees in the Taiyuan Formation limestone karst aquifer, indicating that the deeper karst groundwater was also affected by anthropogenic contaminant infiltration. This source pattern is consistent with the MI results, which showed that heavy metal pollution levels in the Aquifer IV groundwater were lower than those in the Taiyuan Formation limestone karst groundwater.

### 3.4. Health Risk Characteristics and Key Risk Factor Identification

**Figure 5:**
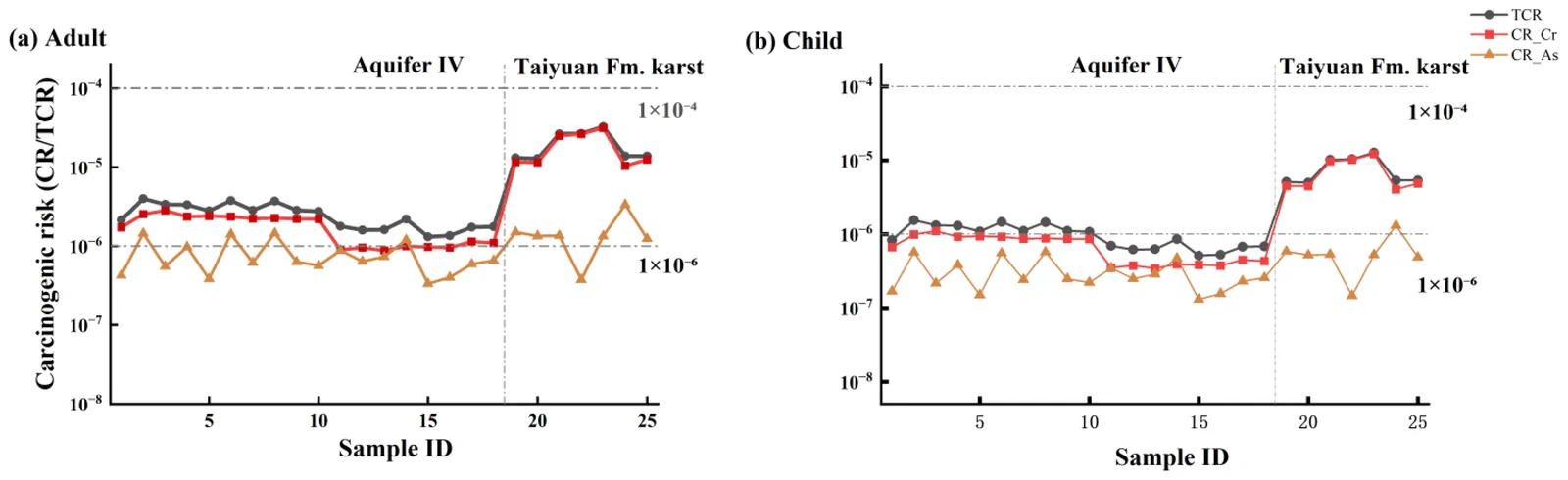
Trajectory variations in total carcinogenic risk (TCR) and the carcinogenic risks of Cr (CR_Cr) and As (CR_As) along the spatial sampling sequence: (**a**) adults; (**b**) children.

Potential health risks from heavy metals in Zhuxianzhuang Coal Mine groundwater were further evaluated with the USEPA oral-ingestion model for drinking water. As and Cr were included in the carcinogenic assessment because they are recognized carcinogenic elements. The single-element carcinogenic risk and total carcinogenic risk (TCR) were calculated, as shown in Figure 5, while non-carcinogenic risk was assessed for the 14 trace elements listed in Table S1.

The non-carcinogenic risk results showed that groundwater in the study area may pose potential non-carcinogenic risks to local residents, with higher risks for children than for adults (Table 3). For children, the mean HI of the Aquifer IV groundwater was 1.26, and 72.20% of the samples had HI values greater than 1. The mean HI of the Taiyuan Formation limestone karst groundwater was 2.15, and all samples exceeded the safety threshold, indicating a higher risk level than that of the Aquifer IV groundwater. For adults, the mean HI of the Aquifer IV groundwater was 0.72, with no samples exceeding the threshold. In contrast, the mean HI of the Taiyuan Formation limestone karst groundwater was 1.23, and all samples exceeded the threshold. Overall, the non-carcinogenic risks of Taiyuan Formation limestone karst groundwater were higher than those of Aquifer IV groundwater for both children and adults, which is consistent with the higher heavy metal pollution levels observed in the Taiyuan Formation limestone aquifer.

The risk contribution results in Table 3 show that As was the primary contributor to non-carcinogenic risk in both groundwater groups, with contribution rates ranging from 39.5% to 48.4%. Sr was the secondary contributor in the Aquifer IV groundwater, whereas Co was the secondary contributor in the Taiyuan Formation limestone karst aquifer. The cumulative contributions of the two dominant factors exceeded 60%, indicating that As was the main controlling factor for non-carcinogenic health risk in groundwater in the study area. Because the assessment considered oral ingestion only, the relative contributions of individual HQ values to HI were broadly similar for children and adults, whereas the main difference between the two receptor groups was in the absolute magnitude of HI.

The carcinogenic risk results showed that the risk curves for adults and children exhibited similar spatial variation patterns. In the carcinogenic risk assessment, the exposure duration for adults was much longer than that for children; therefore, the overall carcinogenic risk level was higher for adults. The TCR values for adults ranged from 1.31 × 10^−6^ to 3.25 × 10^−5^, with a mean value of 7.35 × 10^−6^. For children, the TCR values ranged from 5.11 × 10^−7^ to 1.27 × 10^−5^, with a mean value of 2.87 × 10^−6^. For most samples, TCR exceeded the recommended threshold of 1.0 × 10^−6^, suggesting a potential carcinogenic risk associated with long-term groundwater consumption in the study area [55].

The spatial distribution of carcinogenic risk showed a clear aquifer-related pattern. As shown in Figure 5, the TCR curves for the Aquifer IV groundwater, represented by samples 1–18, fluctuated slightly and remained at a relatively low risk level. After sample 19, corresponding to the Taiyuan Formation limestone karst aquifer, the TCR values increased and remained mostly at the 10^−5^ level. Further comparison of the representative carcinogenic elements showed that, for both receptor groups, the CR-As values generally remained at the low-risk level of 10^−7^–10^−8^. In contrast, the CR-Cr curves were highly consistent with the overall TCR curves, especially after sample 19, indicating that Cr was the main controlling factor for carcinogenic health risk in groundwater in the study area.

The As-dominated non-carcinogenic risk and Cr-dominated carcinogenic risk in the Taiyuan Formation limestone aquifer are consistent with the pollution assessment and source apportionment results discussed above. The MI results indicated that the heavy metal pollution level in Aquifer IV groundwater was lower than that in the Taiyuan Formation limestone karst groundwater. In addition, the PMF results in Section 3.3 identified Cr and As as mainly associated with the combined anthropogenic and mining-related source, represented by Factor 2. This pattern may be related to faults and structural fractures, mining-induced fractures, floor fractures, and water-conducting boreholes in the deeper limestone system, which may facilitate the migration of contaminants into the groundwater flow system and promote local accumulation. In contrast, the thick clay-rich third aquitard in the Cenozoic unconsolidated sequence has low permeability and good confining capacity, which may retard vertical groundwater exchange and associated solute migration. These differences in aquifer media and hydrogeological conditions may explain why the Carboniferous Taiyuan Formation limestone karst groundwater showed more pronounced heavy metal enrichment and carcinogenic risk than Aquifer IV groundwater.

### 3.5. Study Limitations

One limitation concerns the interpretation of the protective role of the clay-rich third aquitard. In this study, this role was inferred from its documented lithology, low permeability, and confining capacity, whereas direct mineralogical characterization and adsorption experiments were not conducted. Therefore, the specific mineral phases responsible for trace-metal retention and the magnitude of sorption cannot be quantified here. Future work combining mineralogical analysis with adsorption experiments would help to further verify the attenuation mechanisms of the clay-rich aquitard.

## 4. Conclusions

This study investigated Aquifer IV groundwater from the Cenozoic unconsolidated porous aquifer system and the Carboniferous Taiyuan Formation limestone karst aquifer in the Zhuxianzhuang Coal Mine. The HPI and MI indices, PMF source apportionment model, and USEPA oral ingestion risk model were used to analyze the distribution characteristics, sources, and health risks of trace elements and heavy metals in groundwater. The main conclusions are as follows:

1. The Taiyuan Formation limestone karst groundwater showed more pronounced elemental enrichment and cumulative contamination effects. Although the HPI results indicated that the overall groundwater quality was within the safe range, the MI, which is more sensitive to cumulative effects, identified more evident combined contamination in the Taiyuan Formation limestone karst groundwater.
2. Combined anthropogenic inputs related to mining and industrial activities were an important source contributing to groundwater contamination in the study area. The PMF model identified two major source factors for heavy metals in groundwater: an Mn-dominated natural geogenic source related to water–rock interaction and a combined anthropogenic source associated with mining and industrial activities and characterized by toxic elements such as As, Pb, and Cr. Sample-level factor contributions further indicated relatively higher Factor 2 contributions in Aquifer IV groundwater and markedly stronger Factor 1 contributions in the Taiyuan Formation limestone karst groundwater. The accumulation of anthropogenic contaminants in the Taiyuan Formation limestone karst aquifer may be an important reason for the higher pollution level in Taiyuan Formation limestone karst groundwater than in Aquifer IV groundwater.
3. As and Cr were the main health risk factors in groundwater in the study area. The total carcinogenic risk (TCR) showed similar spatial variation patterns for adults and children, with higher risks for adults than for children. In contrast, non-carcinogenic risks were higher in the Taiyuan Formation limestone karst groundwater than in the Aquifer IV groundwater, and children showed higher non-carcinogenic risks than adults in both groundwater groups.
4. Differences in aquifer media and structural conditions were important factors affecting pollution levels and health risks between the two groundwater groups. The thick clay-rich third aquitard in the Cenozoic unconsolidated sequence has low permeability and good confining capacity, which may enhance pollutant attenuation by restricting vertical groundwater exchange and solute migration. In contrast, faults and structural fractures, mining-induced fractures, floor fractures, and water-conducting boreholes in the deeper limestone system may facilitate contaminant migration into groundwater and promote local accumulation.

## Figures and Tables

**Table 1:** Exposure parameters utilized for the human health risk assessment.

Parameter	Symbol	Unit	Child	Adult	Reference
Ingestion rate of drinking water (non-carcinogenic)	*IR*	L/d	0.7	1.5	MEP (2013) [34]
Ingestion rate of drinking water (carcinogenic)	*IR*	L/d	1.0	2.0	USEPA; MEP (2013) [34,35]
Exposure frequency	*EF*	days/yr	350	350	USEPA [35]
Exposure duration	*ED*	years	6	30	USEPA [35]
Body weight (non-carcinogenic)	*BW*	kg	16.2	60.6	MEP (2013) [34]
Body weight (carcinogenic)	*BW*	kg	15	70	MEP (2013) [34]
Averaging time (carcinogenic)	*AT_ca_*	days	25,550	25,550	USEPA [35]

**Table 2:** Statistical summary of heavy metal contamination characteristics within distinct aquifers.

Element	Standard Limit (μg/L)	Exceedance Rate (%)	Aquifer IV			Taiyuan Fm. Karst			Mean Ratio (Taiyuan/Aquifer IV)
-	-	-	Range	Mean	CV	Range	Mean	CV	-
As	10	0	2.52~6.18	4.22	0.33	4.45~7.38	5.89	0.20	1.39
Pb	50	0	0.02~0.09	0.05	0.49	0.02~0.2	0.09	0.60	1.95
Sb	5	0	0.18~3.81	1.14	0.73	0.26~2.05	1.13	0.64	0.99
Cr	50	0	5.77~8.83	7.04	0.13	10.71~12.31	11.33	0.04	1.61
Co	50	0	0.16~0.52	0.31	0.41	1.9~5.69	3.35	0.48	**10.63**
Mo	100	0	0.42~1.92	1.20	0.58	1.78~5.07	3.01	0.40	2.52
Mn	100	24	21.75~44.77	32.19	0.23	78.77~519.18	286.06	0.72	**8.89**
Fe	300	0	0.17~6.87	2.23	0.71	1.92~149.79	53.96	1.06	**24.23**
Ba	1000	0	17.93~36.25	26.69	0.26	17.69~37.47	24.55	0.29	0.92
Cu	1000	0	1.4~2.67	1.98	0.21	1.97~2.3	2.14	0.05	1.08
Zn	1000	0	1.8~9.66	4.49	0.54	3.94~10.03	6.50	0.33	1.45
U	30	0	0.06~0.95	0.51	0.80	0.78~1.67	1.23	0.31	2.40
V	50	0	1.81~2.77	2.16	0.14	3.42~3.78	3.56	0.03	1.65
Rb	-	0	21.56~40.28	31.19	0.25	22.19~24.38	23.59	0.03	0.76
Sn	-	0	0~0.01	0.01	0.91	0~0.11	0.02	1.80	3.99
Sr	4000	96	3647.48~5152.06	4490.37	0.07	4717.07~5184.08	4957.37	0.03	1.10

Note: Concentrations and standard limits are expressed in μg/L. Bold values in the Taiyuan/Aquifer IV column indicate the three largest ratios of mean concentrations between Taiyuan Formation limestone karst groundwater and Aquifer IV groundwater.

**Table 3:** Statistical characteristics and primary contributing factors of groundwater non-carcinogenic risks across different populations and aquifers.

Receptor	Aquifer	No. of Samples	HI Range	Mean HI	No. of Samples (HI > 1)	Exceedance Rate (%)	Primary Contributor (%)	Secondary Contributor (%)	Cumulative Contribution of Top 2 (%)
Children	Aquifer IV	18	0.86–1.70	1.26	13	72.20%	As/48.4%	Sr/25.7%	74.1%
	Taiyuan Fm. karst	7	1.96–2.48	2.15	7	100%	As/39.5%	Co/22.4%	61.9%
	Total	25	0.86–2.48	1.51	20	80%	As/44.8%	Sr/22.1%	66.9%
Adults	Aquifer IV	18	0.49–0.97	0.72	0	0	As/48.4%	Sr/25.7%	74.1%
	Taiyuan Fm. karst	7	1.12–1.42	1.23	7	100%	As/39.5%	Co/22.4%	61.9%
	Total	25	0.49–1.42	0.86	7	28%	As/44.8%	Sr/22.1%	66.9%

## Data Availability

The data presented in this study are available on request from the corresponding author due to confidentiality restrictions.

## Supplementary Materials

The following supporting information can be downloaded at: https://www.mdpi.com/article/10.3390/toxics14090740/s1, Table S1, Toxicological parameters used in the non-carcinogenic and carcinogenic health risk assessments; Table S2, Trace-element concentrations in the 25 groundwater samples from Aquifer IV and the Taiyuan Formation limestone karst aquifer (μg/L); Figure S1, Sample-level normalized contributions of Factor 1 and Factor 2 derived from the G matrix of the retained 10-species two-factor PMF solution.
