# Supplementary material for: Source Apportionment and Health Risks of Toxic Metals in Groundwater from a Dual-Aquifer Coal Mine System
Source: Toxics. 2026 Aug 22;14(9):740. doi: 10.3390/toxics14090740 (PMC13611344; doi:10.3390/toxics14090740)
Supplement: Supplementary file 1 [file toxics-14-00740-s001.zip › toxics-4458307-supplementary.pdf]

Supplementary Materials

Source Apportionment and Health Risks of Toxic Metals in Groundwater from a Dual-Aquifer Coal Mine System

Gan Jiang, Kai Chen, and Qimeng Liu

Table S1. Toxicological parameters used in the non-carcinogenic and carcinogenic health risk assessments.

Element	RfD (mg kg^{-1} day^{-1})	RfD source / basis	SF [(mg kg^{-1} day^{-1})$^{-1}$]	SF source / basis
As	3.0×10^{-4}	U.S. EPA IRIS (historical noncancer assessment, 1991)	1.5	U.S. EPA IRIS (historical cancer assessment, 1995)
Sb	4.0×10^{-4}	U.S. EPA IRIS (1987)	—	—
Cr	3.0×10^{-3}	U.S. EPA IRIS (Cr(VI), 1998)	0.5	OEHHA (Cr(VI), 2011)
Co	3.0×10^{-4}	U.S. EPA PPRTV (2008)	—	—
Mo	5.0×10^{-3}	U.S. EPA IRIS (1992)	—	—
Mn	1.4×10^{-1}	U.S. EPA IRIS (1995)	—	—
Fe	7.0×10^{-1}	U.S. EPA PPRTV (2006)	—	—
Ba	2.0×10^{-1}	U.S. EPA IRIS (2005)	—	—
Cu	4.0×10^{-2}	U.S. EPA RSL (derived from HEAST drinking-water value)	—	—
Zn	3.0×10^{-1}	U.S. EPA IRIS (2005)	—	—
U	3.0×10^{-3}	U.S. EPA IRIS (uranium, soluble salts; 1989)	—	—
V	5.0×10^{-3}	U.S. EPA RSL (derived from IRIS V_2O_5 RfD)	—	—
Sn	6.0×10^{-1}	U.S. EPA HEAST (Tin and Compounds)	—	—
Sr	6.0×10^{-1}	U.S. EPA IRIS (1992)	—	—

Note 1. RfDs were used only for non-carcinogenic risks (HQ and HI), whereas SFs were used only for carcinogenic risks (CR and TCR).

Note 2. The values listed reproduce the toxicological parameters actually used in the HRA and TCR calculations of this study.

Note 3. Chromium speciation was not determined; measured total Cr was conservatively evaluated using Cr(VI) toxicity parameters.

Note 4. "—" indicates that no cancer slope factor was used in this study.

Table S2. Trace-element concentrations in the 25 groundwater samples from Aquifer IV and the Taiyuan Formation limestone karst aquifer (μg/L).

Sample ID	Type	V	Cr	Co	Cu	Zn	As	Rb	Mo	Sn	Sb	Pb	U	Mn	Fe	Sr	Ba
S17071	AIV	1.879	5.766	0.313	1.402	1.802	3.912	30.901	1.617	0.003	0.621	0.026	0.916	23.457	4.092	3647.48	29.328
S17072	AIV	2.773	8.834	0.463	1.795	7.408	5.874	37.838	1.92	0.01	1.151	0.088	0.946	41.111	2.263	4587.472	36.245
S17073	AIV	2.566	7.694	0.516	1.822	2.262	5.729	37.729	1.917	0.008	0.461	0.033	0.874	26.016	3.286	4486.488	32.683
S17074	AIV	2.546	8.227	0.432	1.901	6.599	5.841	38.34	1.909	0.012	1.13	0.059	0.793	24.532	4.199	4390.136	31.567
S17075	AIV	2.661	8.703	0.44	1.651	2.183	6.177	39.057	1.917	0	1.9	0.023	0.828	25.431	2.713	4757.027	33.807
S17076	AIV	2.387	7.912	0.432	1.752	7.385	5.589	38.622	1.707	0.001	1.087	0.086	0.861	29.544	1.029	4611.861	33.215
S17077	AIV	2.263	7.28	0.406	1.507	4.214	5.41	38.203	1.74	--	1.925	0.038	0.851	25.888	0.667	4602.134	32.868
S17078	AIV	2.063	6.565	0.412	1.541	9.665	5.063	38.726	1.711	0.002	1.255	0.089	0.853	25.728	1.479	4596.896	34.094
S17079	AIV	1.948	6.456	0.403	1.459	4.651	4.851	38.073	1.68	0	1.166	0.038	0.847	26.147	0.167	4462.369	31.609
S170710	AIV	1.994	6.674	0.402	1.565	3.071	5.163	40.277	1.773	--	1.067	0.034	0.924	21.755	1.804	4764.936	31.523
S170711	AIV	1.983	6.97	0.164	2.496	2.963	2.521	23.058	0.466	--	0.26	0.054	0.066	40.88	1.919	4584.321	19.315
S170712	AIV	1.954	6.23	0.174	2.408	3.664	2.606	23.075	0.481	--	0.943	0.039	0.067	34.305	1.017	4529.919	19.462
S170713	AIV	1.813	6.316	0.16	2.341	2.735	2.644	21.779	0.444	0.012	0.179	0.044	0.061	36.349	1.489	4220.899	17.925
S170714	AIV	1.994	6.318	0.182	2.418	8.665	2.892	23.32	0.469	0.008	3.812	0.073	0.069	39.281	1.74	4334.058	19.062
S170715	AIV	1.939	6.731	0.179	2.361	2.791	2.852	22.453	0.437	--	0.559	0.02	0.065	38.139	6.868	4477.368	19.116
S170716	AIV	1.836	6.356	0.175	2.204	2.405	2.683	21.563	0.423	--	0.49	0.024	0.068	36.838	2.051	4144.655	18.077
S170717	AIV	2.189	7.055	0.209	2.672	3.074	3.31	25.867	0.487	0	0.886	0.036	0.079	44.771	1.798	5152.06	21.03
S170718	AIV	2.034	6.699	0.201	2.363	5.197	2.904	22.485	0.422	0.009	1.71	0.04	0.077	39.226	1.511	4476.51	19.581
S170719	TLK	3.78	12.31	2.12	2.302	3.939	7.376	23.961	1.804	0.108	0.259	0.091	1.436	121.817	103.65	5184.077	21.969
S170720	TLK	3.584	11.5	2.095	1.972	7.945	6.615	23.458	1.785	0.006	2.047	0.081	1.409	140.073	149.793	5017.778	21.414
S170721	TLK	3.471	11.242	4.544	2.169	7.035	4.448	23.78	3.795	0.001	0.615	0.083	0.784	486.565	4.37	4717.072	23.877
S170722	TLK	3.478	11.039	4.79	2.036	4.095	4.752	24.308	3.522	0.016	0.435	0.023	0.836	519.185	1.925	4960.571	18.556
S170723	TLK	3.416	10.708	5.687	2.184	10.03	4.84	24.381	5.071	0.01	1.841	0.082	0.883	508.879	2.27	4962.252	17.691
S170724	TLK	3.606	11.103	1.899	2.166	6.543	6.547	23.08	2.48	0.004	1.064	0.204	1.601	78.773	67.772	4927.333	30.884
S170725	TLK	3.559	11.395	2.285	2.164	5.916	6.626	22.187	2.633	0.004	1.676	0.076	1.667	147.132	47.956	4932.474	37.466

Note. AIV = Aquifer IV groundwater; TLK = Taiyuan Formation limestone karst groundwater. All concentration values are reproduced directly from the original analytical dataset. "--" denotes a value reported as below the detection limit in the original dataset; values reported as 0 are retained unchanged.

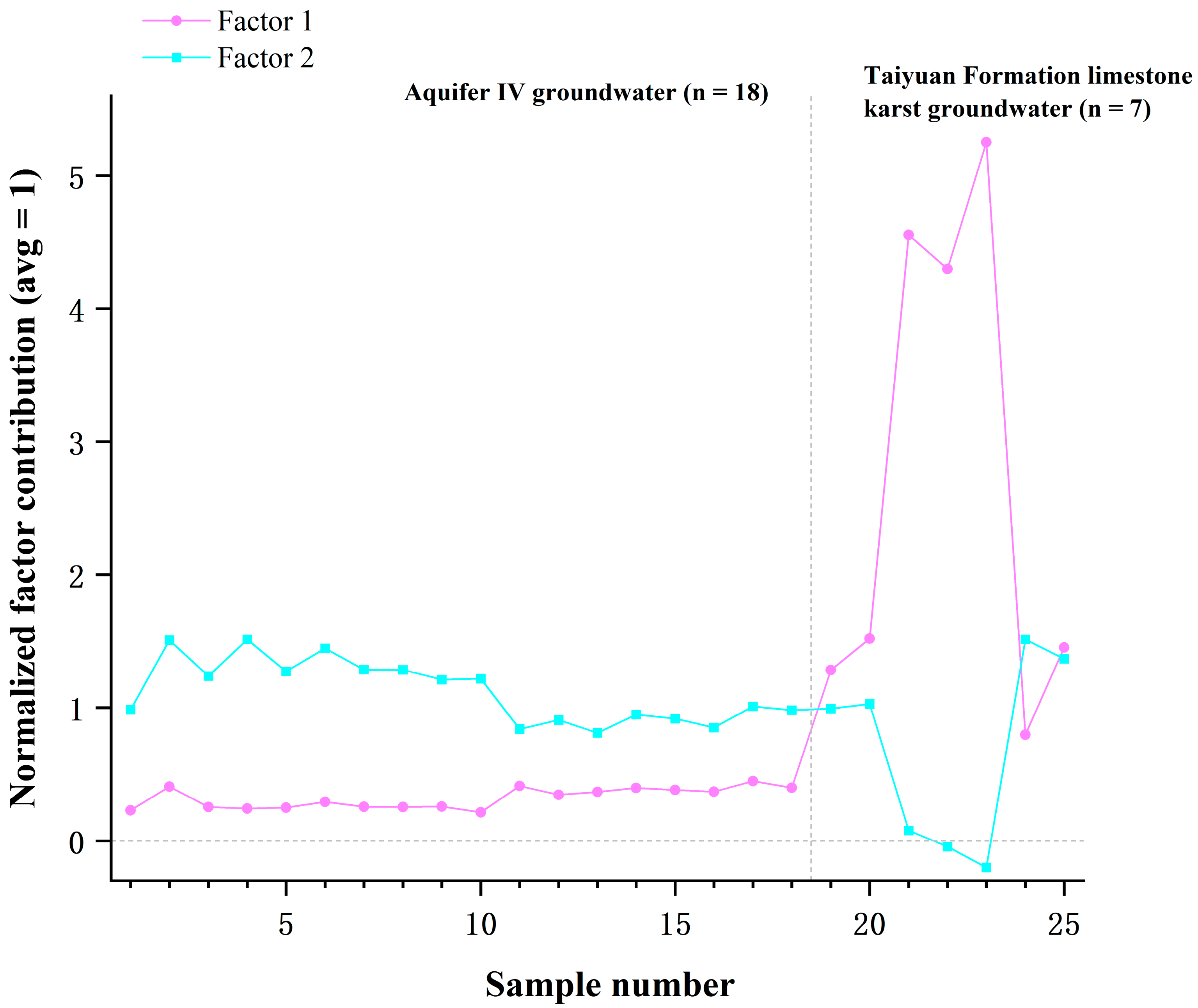

Figure S1. Sample-level normalized contributions of Factor 1 and Factor 2 derived from the G matrix of the retained 10-species two-factor PMF solution.

Note. Samples 1–18 represent Aquifer IV groundwater, whereas samples 19–25 represent Taiyuan Formation limestone karst groundwater. Factor contributions are normalized to an average value of 1 in EPA PMF 5.0.
